# In Vitro and In Vivo Evaluation of Poly (3-hydroxybutyrate)/Carbon Nanotubes Electrospun Scaffolds for Periodontal Ligament Tissue Engineering

**DOI:** 10.30476/DENTJODS.2019.77869

**Published:** 2020-03

**Authors:** Moein Zarei, Saeed Karbasi, Fatemeh Sari Aslani, Shahrokh Zare, Omid Koohi-Hosseinabad, Nader Tanideh

**Affiliations:** 1 Stem Cells Technology Research Center, Shiraz University of Medical Sciences, Shiraz, Iran; 2 Dept. of Biomaterials and Tissue Engineering, School of Advance Technology in Medicine, Isfahan University of Medical Sciences, Isfahan, Iran; 3 Molecular Dermatology Research Center, Dept. of Pathology, Shiraz University of Medical Sciences, Shiraz, Iran; 4 Central Research Laboratory, Shiraz University of Medical Sciences, Shiraz, Iran; 5 Stem Cells Technology Research Center, Dept. of Pharmacology, Shiraz University of Medical Sciences, Shiraz, Iran

**Keywords:** Tissue engineering, Poly (3-hydroxybutyrate), Multi-Walled Carbon nanotubes, Scaffold, In vitro, In vivo, Periodontal regeneration

## Abstract

**Statement of the Problem:**

Tissue engineering was an idea, today it has become a potential therapy for several tissues in dentistry, such as periodontal disease and oral mucosa.

**Purpose:**

In this experimental study, periodontal regeneration is one of the earliest clinical disciplines that has achieved therapeutic application in tissue engineering. The aim of the present study was to prepare electrospun Poly (3-hydroxybutyrate) (PHB)/1% Carbon nanotubes (CNTs) scaffolds for periodontal regeneration.

**Materials and Method:**

1% w/v of CNTs was added to the polymer solutions and electrospinned. Physical properties of the scaffolds were evaluated by scanning electron microscopy (SEM) and universal testing machine. Chemical characterization of the scaffolds was also assessed by Fourier-transform infrared spectroscopy (FTIR). Biological properties of the scaffolds were also evaluated in vitro by culturing periodontal ligament stem cells (PDLSCs) on the scaffolds for 10 days and in vivo by Implanting the scaffolds in rat model for 5 weeks.

**Results:**

Results showed that the scaffolds mimicked fibrous connective tissue of the (PDL). CNTs improved the mechanical properties, similar to 23-55 years old human PDL. In vitro biocompatibility study showed more attachment and proliferation of the PDLSCs for PHB/1%CNTs scaffolds compared to the PHB controls. In vivo study showed that CNTs in the scaffolds caused mild foreign body type giant cell reaction, moderate vascularization, and mild inflammation.

**Conclusion:**

The results showed that the PHB/1%CNTs composite scaffolds might be potentially useful in periodontal regeneration.

## Introduction

The periodontal ligament (PDL) is a soft tissue embedded between the cementum (a thin layer of mineralized tissue covering the roots of the teeth) and the inner wall of the alveolar bone socket, to sustain and help constrain the teeth within the jaw [ 1 ]. The PDL is composed of fibrous connective tissue and a physically small but functionally important tissue in tooth support, proprioception and regulation of alveolar bone volume [ 2 ]. Periodontal diseases are the most common inflammatory diseases caused by plaque biofilm in the oral cavity. For more than three decades, periodontal research has attempted to discover clinical treatment regimens that can regenerate periodontal tissues with good predictability [ 3 ]. The strategy aimed at regenerating a 3D-arrayed structure of lost PDL tissue with the connective tissue attachment. To achieve complete regeneration, the introduction of modern tissue engineering technology is anticipated in this field [ 3 ]. Tissue engineering combines the principles and methods of the life science with those of engineering to elucidate fundamental understanding of structure-function relationships in the normal and diseased tissues, to develop materials and methods to repair damaged or diseased tissue, and to create entire tissue replacements [ 4 ].

 In this context, three-dimensional porous scaffolds can provide the condition, necessary for the cells to proliferate, migrate, and maintain their differentiated function by creating an empty space and function as a framework for thriving tissue [ 5 - 6 ]. Myriad natural and synthetic materials have been evaluated for tissue engineering scaffolds [ 6 ]. The sizes of extracellular matrix components, including porosities and the fibers diameters are at a range of nano-scales, using nano-fibers has increased in tissue engineering [ 7 - 8 ]. Amongst numerous methods, electrospinning process is considered a cheap method to prepare nanofibers from both natural and synthetic materials (fiber diameters from micrometer to nanometer range) [ 9 - 10 ]. Electrospun scaffolds offer an appropriate morphology and porosity that resemble the natural extracellular matrix (ECM) by providing a good condition for cell attachment, proliferation, and differentiation [ 11 - 12 ]. 

In the past decades, PHB is considered as a biodegradable and biocompatible material that has been applied for biological applications in medicine [ 13 ]. The challenging between combination of biomedical and biodegradable properties of PHB is a perspective tool in the design of novel medical devices and tissue engineering [ 14 ]. Although this polymer has a myriad of medicinal application and the properties necessary for use as a scaffold, it does not have enough strength, which is necessary for the three-dimensional scaffolds, for hard tissue engineering [ 15 ]. Selection of a suitable material to improve the mechanical and biological properties of PHB would be imperative. Regarding this issue, carbon nanotubes have been extensively used for biological and biomedical applications in the past few years due to their unique intrinsic physical, chemical, and mechanical properties [ 16 ]. Evaluations of many researchers have shown that functionalized CNTs are able to enter the cells without toxicity, shuttling various biological molecular cargoes into the cells [ 17 - 18 ]. Despite these exciting findings, researchers reported the negative sides of CNTs, including those non-functionalized nanotubes are toxic to the cells and animals [ 17 ]. CNTs functionalization is thus, required and involves the addition of functional groups such as carboxyl or alcohol groups to the walls and ends of the nanotubes [ 18 ]. This should prevent CNTs aggregation and allow for their incorporation into polymer scaffolds [ 19 ]. Studies showed that nanoparticles of bioceramics could improve the properties of the polymers both mechanically and biologically. For significant increase in the properties of the polymers by nanoparticles, high amounts of nanoparticles (Close to 10-20% by weight) have to be added to the basic polymer [ 20 - 21 ]. However, numerous studies showed that CNTs with low percentages (Close to 1-2% by weight) could significantly increase the properties of different polymers [ 22 - 23 ]. Jeong *et al*. [ 22 ] evaluated different amounts of CNTs (1%, 2.5%, 5%, and 7.5%) on polyvinyl alcohol scaffolds and revealed that 1% of the CNTs had the most improvement in mechanical properties of the scaffolds. In another study, the results indicated that the presence of only 0.5% of functionalized CNTs in poly-lactic glycolic acid scaffolds increased the tensile strength by 54% and increased the in vitro cell compatibility compared with the poly-lactic glycolic acid control [ 24 ]. 

Regarding the biological and mechanical properties of functionalized CNTs and using them as confirmatory material in the scaffolds, the aim of the present study was to prepare PHB/1%CNTs nanocomposite scaffolds via electrospinning and evaluate their structural, mechanical properties (according to the PDL), in vitro cell compatibility, using human PDLSCs and in vivo tissue biocompatibility (according to the tissue engineering parameters) for oral tissue engineering applications.

## Materials and Method

### Materials

Poly (3-hydroxybutirate) was purchased from Sigma-Aldrich Inc, USA. Functionalized multi-walled carbon nano-tubes (COOH) measuring 5‒25 nm in diameter, 0.5‒2 µm in length and purity >95wt% were purchased from US Research Nanomaterials, USA. Dimethyl formamide (DMF), trichloromethane (TCM), mixture of penicillin and streptomycin and dimethyl sulfoxide (DMSO) were purchased from MERCK, Germany. Simulated body fluid (SBF) (Nik Ceram Razi, Iran), Dulbecco's Modified Eagle's Medium (a-MEM) and trypsin (GIBCO Life Technologies, Grand Island, NY, USA), fetal bovine serum (FBS), MTT (M-2128, Sigma, St. Louis, MO, USA), and nylon 3–0 suture (Ethicon, Johnson and Johnson, USA) were purchased for this research.

### Electrospinning

The electrospinning equipment used in the present study consisted of a power source, an injection pump, an aluminum plate, a 1mL syringe, and a needle with an internal diameter of 0.27 mm. According to a previously published article, PHB was dissolved in mixed solution of TCM and DMF with the volume ratio of 7/3 for 60 minutes at 50ºC [ 25 ]. 1 wt% of functionalized CNTs was added to the PHB solution and the mixture was ultrasonicated for 1 h. The PHB and PHB/1%CNTs solution was separately loaded into the 1mL syringe with the needle attached. The needle was pointed toward the aluminum plate and a constant positive voltage (12.5 kV) was slowly applied to the needle. The distance between the needle tip and the aluminum plate was 25 cm and the syringe pump was set at a flow rate of 0.01 mL/min. A fluid jet was formed from the needle and fibers were drawn onto the aluminum foil to collect the fibers. All experiments were carried out in the air at 25ᵒC and 60% relative humidity.

### Structural characterization 

Based on the required structural specifications for the scaffold systems, and in order to prove the presence of CNTs in the scaffolds, we separately evaluated the morphology, porosity, and changes in the chemical structure of the scaffolds by different methods. VEGA3/Tescan scanning electron microscope (SEM) was applied to evaluate the surface structure and effect of CNTs in the structure of fibers. MATLAB software program can easily be used to measure the porosity of various layers via SEM photomicrograph [ 26 ]. To measure the porosity, 5 different SEM images from each group were chosen and evaluated by MATLAB software program. Fourier-transform infrared spectroscopy (FTIR) technique was applied to evaluate the chemical structure and presence of CNTs in the scaffolds.

### Mechanical characterization

Based on ISO 1798 specifications, the mechanical properties of the scaffolds were evaluated by tensile strength test at room temperature. The scaffold in the dimension of 10-50 mm (WL) was supported between two clamping jaws and pulled apart by extension rate at 10 mm/min until the specimen fractured at a constant rate of displacement. The load cell used was 20N with a gauge length of 25 mm. 10 samples for each group were used to evaluate and compare the mechanical properties of the scaffolds.

### Surface wettability 

The contact angle was measured with a drop on the specimen after 1 minute of drop deposition [ 27 ]. The measurements of static contact angles were mainly performed with the specimen placed on a horizontal plane; a drop of water was dropped on the surface of the sample via a precise dropper and the picture of drop on the specimen was seen using a telescope with a calibrated micrometer lens. On each occasion, at least 10 measurements were taken, and the static contact angles were determined using the Image J software.

### Bioactivity evaluation

According to Kokubo and Takadama's definition of bioactivity, a bioactive material is one on which bone-like hydroxyapatite will form selectively after it is immersed in a serum-like solution [ 28 ]. In this study, SBF solution environment was used to characterize the bioactivity of the scaffolds by immersing them into the solution for four weeks. During this period, from the first to the fourth week, 5 samples were used for each group and atomic absorption spectroscopy (AAS) method (AAS-Perkin Elmer Co-A-Analyst-300) was weekly employed to measure the absorption level of Ca2+ in the SBF solution. SEM and energy-dispersive X-ray (EDX) were applied to observe and prove the existence of sedimentary hydroxyapatite crystals on the surface of the fibers. After four weeks, X-ray diffraction (XRD) was also used to study the structure and prove the presence of hydroxyapatite on the surface of the fibers for more assurance.

### PDL cell isolation

In vitro cell culture was performed using PDLSCs derived from human body. The common impacted third molars (n=25) were collected from four 20-22 year old healthy men at the School of Dentistry, Shiraz University of Medical Sciences. PDL was softly and completely separated from the root and then placed in a solution of 3 mg/mL collagenase type I (Gibco) and 4 mg/mL dispase (Gibco) as digestion solution for 1 h at 37°C. Different PDL specimens were combined and single-cell suspensions were achieved by crossing the cells through MS CA Syringe Filter [ 1 ]. Single-cell suspensions (1×104 cells) were seeded into 10-cm culture dishes (JET BIOFIL) with DMEM F12 (SHELL MAX) supplemented with 15% FBS (SHELL MAX), 100 μmol/L ascorbic acid 2-phosphate (SIGMA), 100 U/mL penicillin, and 100 μg/mL streptomycin (SHELL AMX), and then incubated at 37°C in 5% carbon dioxide to recognize putative stem cells. The prepared scaffolds (10 samples for each group) with 6mm in diameter were separately placed in 96-welled tissue culture polystyrene plates (Corning, Action, MA, USA). The scaffolds were sterilized in 70% ethanol overnight and rinsed extensively with phosphate-buffered saline (PBS), followed by treatment under ultraviolet light overnight. Subsequently, 200µL medium of PDLSCs suspension at a concentration of 105 cells/ml was added to each well and followed by incubation for 1, 5 and 10 days, with medium replacement every day. Cell suspension was also placed in empty tissue culture polystyrene dish as the control groups.

### Evaluation of PDL Cell adhesion and proliferation 

Morphological study of PDLSCs grown on electrospun PHB and PHB/1%CNTs was performed by SEM after 1 and 10 days of cell culture. The cell-seeded scaffolds were collected after 1 and 10 days and entirely washed with PBS solution. Subsequently, the cells on the scaffolds were fixed by 2.5% glutaraldehyde in PBS for 1h at 4ᵒC. Afterwards, the specimens were dehydrated using 50-100% ethanol with 30 min each grade, dried, gold splattered in vacuum, and examined using SEM. MTT assay was used to assess the PDLSCs’ metabolic activity in the presence of intended scaffolds. Briefly, MTT solution was ready in PBS, sterilized, added to each well, and incubated for 3h at 37ᵒC. Then, to dissolve the red colored formazan crystals formed, we added dimethyl sulfoxide and the absorbance was measured at 570nm with ELISA plate reader.

### Animal study

Ethical considerations were confirmed by the Animal Care and Use Committee of the Shiraz University of Medical Sciences (Approval No: IR.SUM.REC.1396-S1037). Sprague dawley male rats (aged 8–10 weeks; weighted 220±20g; n= 5 rats for each group) were prepared from Laboratory Animals Center of Shiraz University of Medical Sciences, Shiraz, Iran. All the rats were kept at standard room (temperature (22±2°C); humidity 55±5%; ventilation 12 times per hour and 12 hours light/dark cycle). They were fed a standard pellet diet ad libitum.

### Scaffold implantation

The rats were anesthetized using 90mg/kg Ketamine 10% (Alfasan, woerden-Holand) and 8 mg/kg Xylazine 2% (Alfasan, woerden-Holand) with the eyes protecting the application of ophthalmic liquid gel (Alco Canada In., ON, Canada). The rat’s back hair was shaved and sterilized using Povidone lodine 10% (Pejhan Chemic Yazd Co. Iran), 5mm incisions were cut on the dorsal section of each rat with aseptic surgery method, and cell-free scaffolds were implanted under subcutaneous pouch. The incisions were then sutured and oxytetracycline aerosol spray was topically applied to the surgery sites to prevent infection. Additionally, 0.03 mg/kg buprenorphine was administrated as a pain reliever. All the rats were carefully monitored during the study.

### Histopathological analysis 

The aim of histological assessment was to evaluate the biocompatibility of the scaffolds including extracellular matrix deposition, vascularization, formation of foreign body giant cell, and inflammation. At the end of the 5th week after the surgery, the rats were euthanized using CO2 inhalation in a specific chamber; the dorsal skin was carefully resected and fixed in 10% formalin. Serial 5μm thick sections were cut, and stained with hematoxylin-eosin (H&E). For the evaluation of cell infiltration, extracellular matrix deposition and vascularization (angiogenesis), micrographs were captured using Olympus microscope (model BX53F, Japan) equipped with 40x and 400x objective and evaluated by a blind pathologist.

### Statistical analysis

The collected data were analyzed by one-way analysis of variance (ANOVA) and reported as mean standard deviation. All observations were confirmed by at least three independent experiments. The Data were analyzed using IBM SPSS software and the level of significance was set at *p*< 0.05.

## Results

### Morphological characterization

**Figure1:**
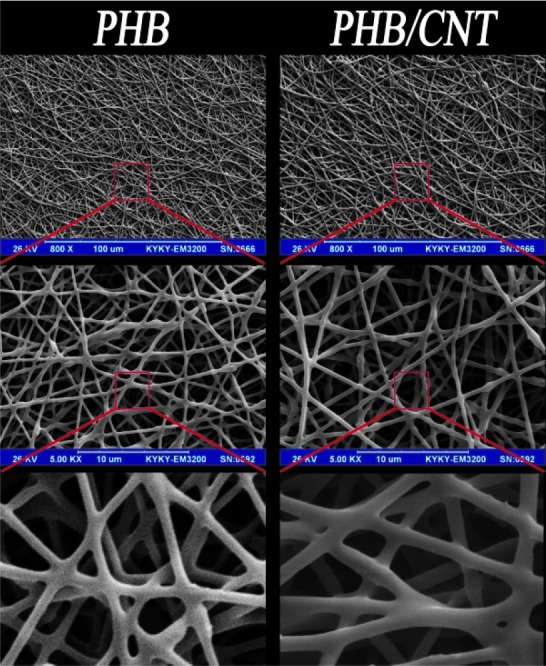
SEM photos of pure PHB scaffold, PHB/1% CNTs scaffold in different scales

**Figure2:**
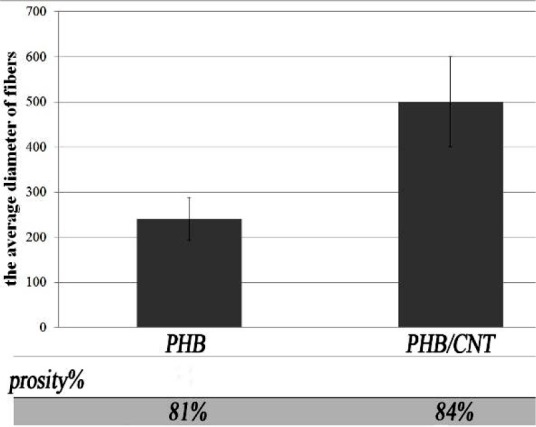
The average diameter and the porosity of PHB and PHB/1%CNTs scaffold according to SEM photos (*p*< 0.05)

Figure 1 illustrates the morphology of pure and composite scaffolds. They mimicked the human PDL tissue (provided by Beertsen *et al*.) [ 29 ]. Presented SEM micrograph of electrospun fibers showed that the created fibers were structurally similar to the collagen fibrous structure in PDL. The obtained fibers of the scaffolds were smooth without any bead; the majority of the fibers’ diameter for pure PHB was confined to the range of 180‒320 nm, with a mean of 240nm (Figure 2). This range of fiber diameter for composite scaffold increased to 400-590nm by addition of only 1% w/v CNTs. 

By using MALAB software program, the obtained results of porosity showed that the porosity of electrospun nano fibrous scaffolds was over 80%, which is suitable for the purposes of tissue engineering. 

### Chemical structure 

**Figure3:**
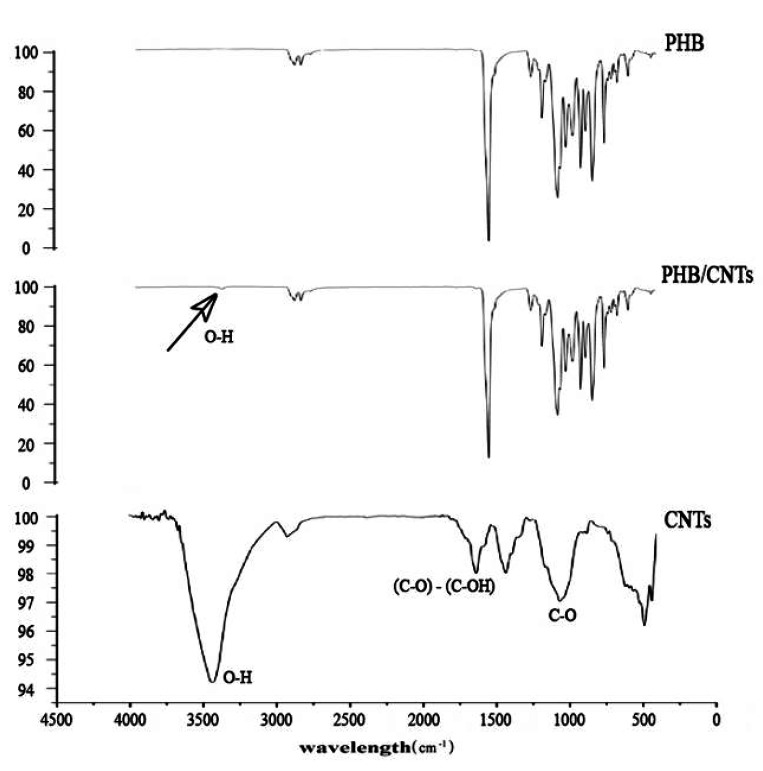
FTIR of pure PHB, PHB/1%CNTs Nanocomposites scaffold and pure CNTs powder

The spectrums obtained from pure PHB and CNTs and the composite scaffold from FTIR technique allow us to identify the structures and bonds and compare them with each other (Figure 3).

In the obtained spectrum of pure PHB, the absorptions were in 970cm^-1^ – 1725cm^-1^ regions, which mainly correspond to the structures and bonds of CH, CH2, CH3, C-O and O-H of PHB. The remarkable peaks in the spectrum of CO-OH functionalized CNTs were O-H bonds (2910cm^-1^ – 3430cm^-1^) and C-O bond (870cm^-1^ – 1150cm^-1^), corresponding to the functional groups that had been situated on the surface of nanotubes. In electrospun nanocomposite scaffold spectrum, although nanotubes surface peak (O-H bond) appeared indiscernibly at around 3430cm^-1^, it verifies the distribution of CNTs in PHB scaffold. The indiscernibility of the O-H peak in composite scaffold is caused by the low quantity of CNTs in composite scaffold.

### Mechanical characterization

**Figure4:**
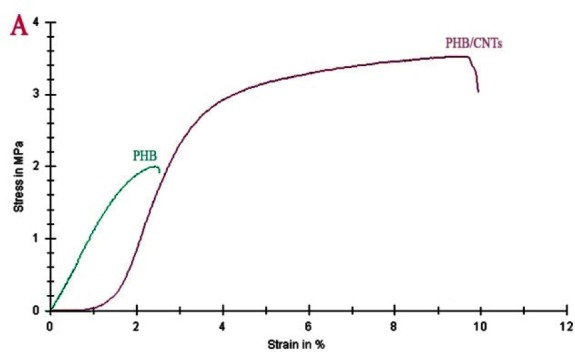
Variations in tensile stress (MPa) as a function of strain (%) for PHB and PHB/1%CNTs scaffold (*p*< 0.05).

The tensile strengths of pure PHB and PHB/1%CNTs are shown in Figure 4. The results showed that the tensile strength of nano-composite scaffold was close to tensile strength of the human PDL compared to pure PHB [ 30 ]. The tensile strength of the composite scaffold compared to pure scaffold clearly and significantly increased in the presence of only 1% CNTs. 

### Water contact angel

**Figure5:**
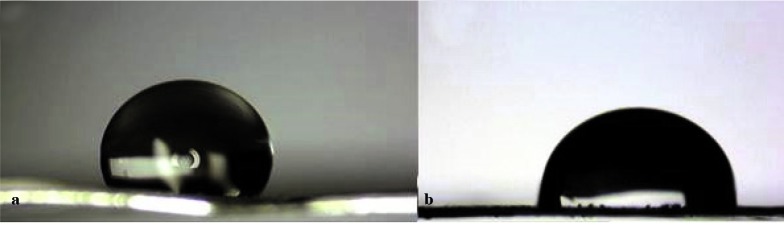
(a) pure PHB and (b) PHB/1%CNTs scaffolds

The macro-photograph of the water drop on pure and composite scaffolds surfaces is presented in Figure 5. One minute after placing the drop on the surface of scaffolds, the contact angles of water drop with the surface of the scaffolds are shown in Table 1. According to the results, the water contact angel (WCA) decreased from 121◦ for pure scaffold to 86◦ for composite scaffold. 

**Table1:** Static contact angle of water drops on the scaffolds after 1 minute

Contact Angel	%CNTs
ᵒ121	0%
ᵒ86	1%

### Bioactivity 

**Figure6:**
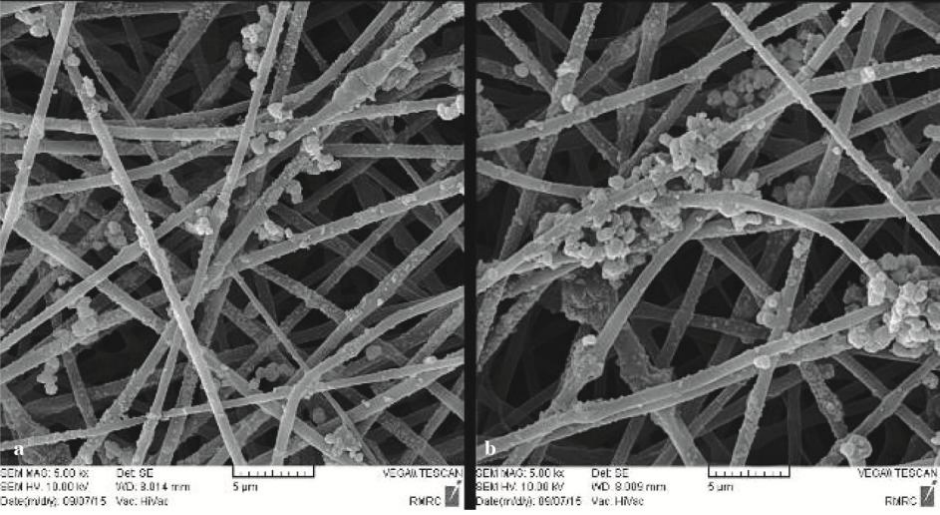
SEM image of sedimentary apatite particles constituent of pure PHB and PHB/1%CNTs scaffolds

**Figure7:**
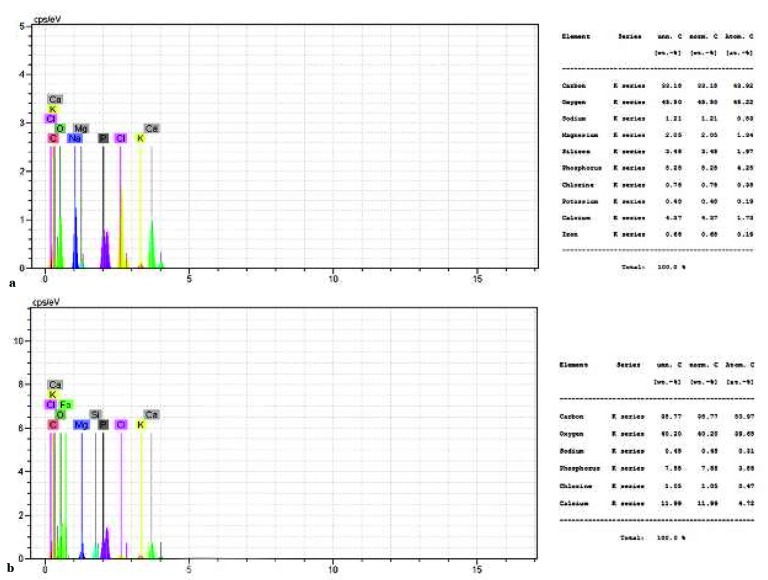
EDX analysis of pure PHB scaffolds (a) and PHB/1% CNTs (b) after 28 days in SBF solution

**Figure8:**
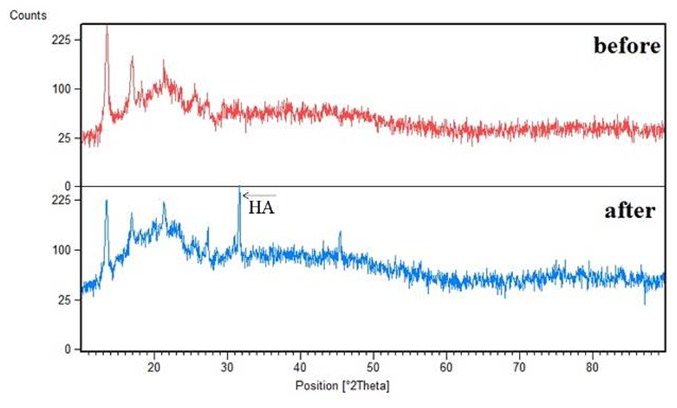
XRD of PHB/1%CNTs (before and after submersion in SBF solution)

To evaluate the bioactivity of the scaffolds, we immersed them in the SBF solution. After 4 weeks from immersion, the SEM images and EDX analysis represented the creation of sedimentary apatite crystals on the surface of nanofibers (Figures 6 and 7). More sedimentary apatite can be observed in the composite nano-fibers compared to pure PHB. The EDX analysis of both pure and composite scaffolds showed the calcium and phosphorus which belonged to the formation of hydroxyapatite. Although the existence of calcium and phosphorous was obtained by EDX analysis, this method cannot be applied to specify the exact chemical composition of the material. However, X-ray diffraction patterns can be employed to specify the exact chemical composition of the hydroxyapatite on the fibers surface. The patterns in Figure 8 represent and compare the XRD of the composite scaffold before and after 4 weeks of bioactivity examination.

**Figure9:**
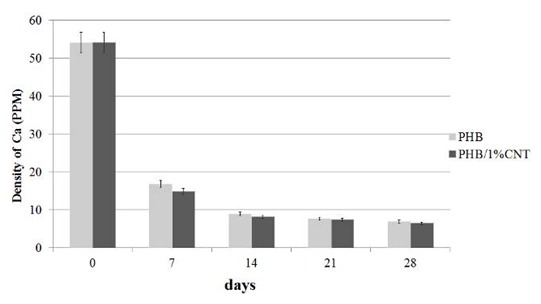
Absorption of calcium ion of SBF solution by scaffolds in the 7th, 14th, 21th and 28^th^ days.

According to the pattern, the obtained peak in the domain of 30ᵒ-35ᵒ corresponded to HA phase. Atomic absorption spectroscopy (AAS) was used to characterize atomic absorption of calcium in SBF solution. Lower amounts of calcium in SBF means more absorption by the material, resulting in more bioactivity. The absorption of calcium in SBF was determined as the control group, which was 34 ppm (in Figure 9). The absorption of calcium for both pure and composite scaffolds decreased to less than 10 ppm after 2 weeks. However, after 4 weeks, the absorption of the composite scaffold was indiscernibly less than pure PHB, which means more bioactivity for the composite scaffold.

### Cell attachment

**Figure10:**
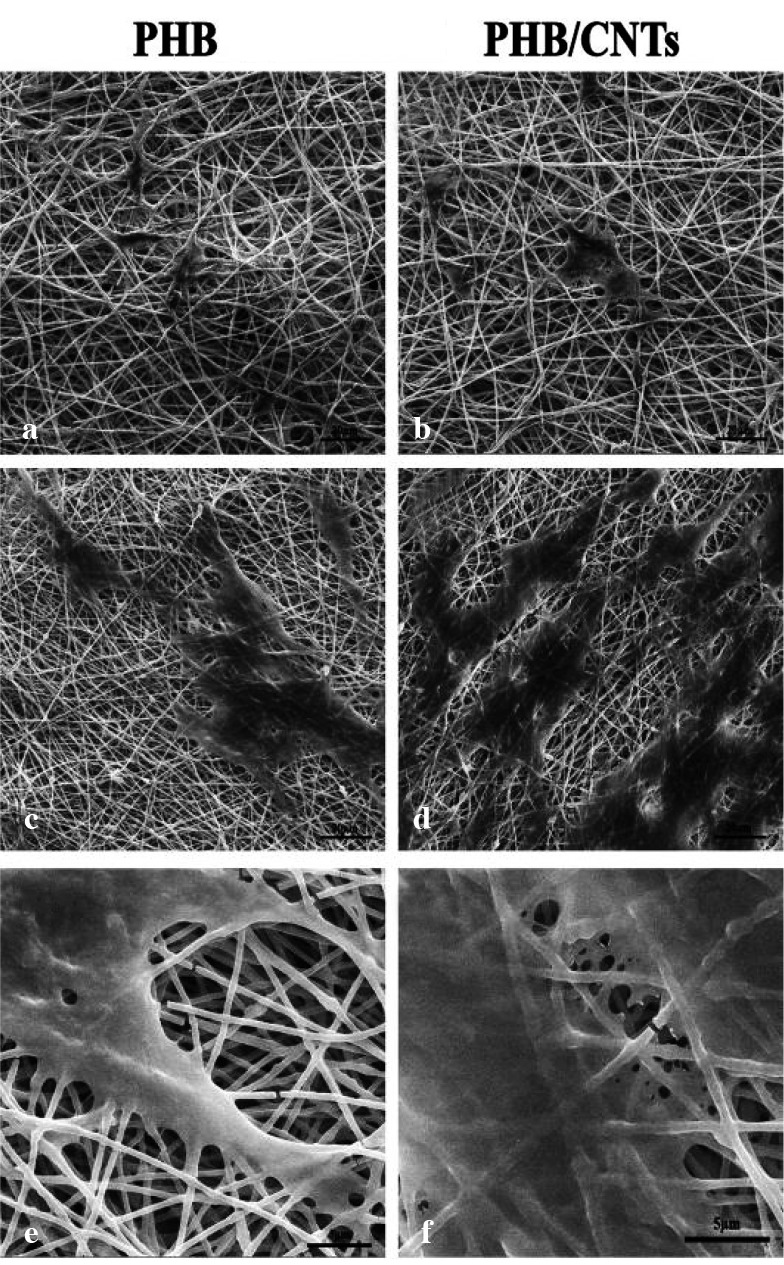
SEM images of PDL cell morphology; **a:** on pure PHB scaffolds on the first day of culture at 1200x magnification, **b:** on PHB/1% CNTs scaffold on the first day of culture at 1200x magnification, **c:** on pure PHB scaffold on the 10th day of culture at 1200x magnification, **d:** on PHB/1% CNTs scaffold on the 10^th^ day of culture at 1200x magnification, **e:** on pure PHB scaffold on the 10th day of culture at 5000x magnification, **f:** on PHB/1%CNTs scaffold on the 10th day of culture at 5000x magnification

According to the microscopic analysis, the cells were spherical and floated before culturing on cell culture dishes but after that, the cells were attached to the bottom of the dishes and they were spindle like. After 1 and 10 days of cell seeding on the scaffolds, the attachment and morphology of PDLSCs on the surface of pure and composite scaffolds was observed and evaluated by SEM (Figure 10). SEM micrographs showed the cell attachment on both pure and composite scaffold. There was more attachment of PDLSCs on the surface of composite scaffold compared to pure scaffold, which can be clearly seen in SEM graphs. 

### MTT assay

In this study, we used MTT assay to assess and compare cell metabolic activities of both pure and composite scaffolds for 10 days.

**Figure11:**
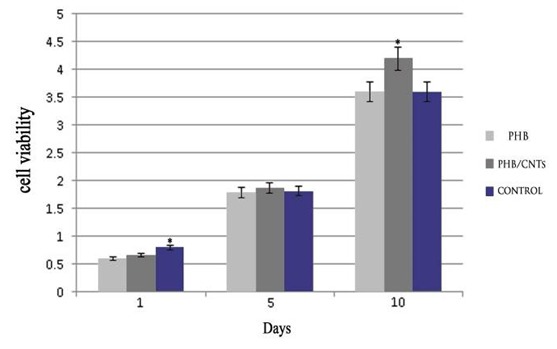
Proliferation of PDLSCs on the electrospun PHB and PHB/1%CNTs evaluated by MTT test on days 1, 5 and 10. Bar represents mean± SD. *p*< 0.05, one-way ANOVA test.

As can be seen in Figure 11, the viability of PDLSCs on the composite scaffold significantly increased compared to pure PHB. However, in comparison to the control group (scaffold-free) after days 1 and 5, no significant differences were observed between pure and composite scaffolds by PDLSCs. The percentage of the viability of the cells on the scaffolds containing 1% CNTs significantly increased after 10 days compared to the pure PHB scaffold and control groups (*p*< 0.05).

### Histopathological assessments

**Figure12:**
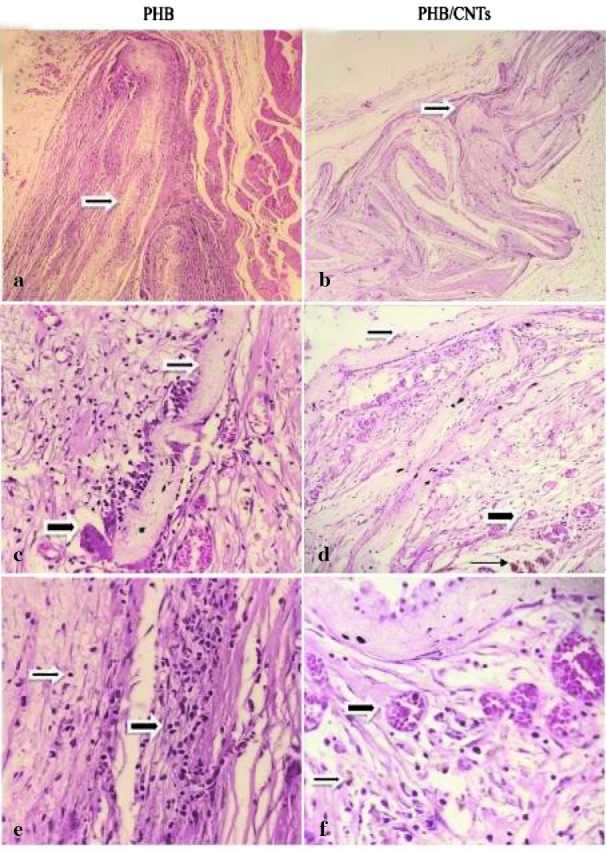
Microscopic sections of the scaffolds within the tissue stained with H&E. **a:** low magnification shows Pure PHB scaffold (thin arrow); **b:** mid magnification shows foreign body type giant cell (thick arrow) in the vicinity of PHB scaffold (thin arrow); **c:** High magnification shows lymphocytic infiltrate ( thin and thick Arrow) within and in the vicinity of scaffold; **d:** low magnification shows PHB/ 1% CNTs scaffold (thin arrow); **e:** mid magnification shows PHB/1% CNTs vascularization (thick arrow), scaffold (thin arrow), hemosiderin laden macrophages (very thin arrow); **f:** High magnification shows vascularization (thick arrow) hemosiderin laden macrophages (thin arrow).

The tissue reaction was subcutaneously characterized at the area between the scaffold and surrounding connective tissue. Observations during the implantation time showed that animals generally have no systematic or neurological toxicity. The observations were based on the absence of distress symptoms, such as reduced activity, weight loss, or immobility. A summary of the histological response of the implanted pure and composite scaffolds is presented in Table 2. Microscopic examination of pure PHB scaffold after 5 weeks of subcutaneous implantation showed moderate foreign body type giant cell reaction in the vicinity of the scaffold, mild angiogenesis (vascularization), moderate inflammatory infiltration composed of predominantly mononuclear cells (lymphocytes, histiocytes), and a few polymorphonuclear cells within and in the vicinity of the scaffold (Figure 12a, b and c). Microscopic examination of PHB/1%CNTs showed mild foreign body type giant cell reaction in the vicinity of scaffolds, moderate angiogenesis (vascularization), mild inflammatory infiltration composed of mononuclear cells (lymphocytes, histiocytes), within and in the vicinity of the scaffolds as well as a few hemosiderin laden macrophages due to mild hemorrhage (Figure 12d, e and f).

**Table2:** A summary of the histological response of implanted pure PHB and PHB/CNTs composite scaffolds. (Mild= Mild, Mod= Moderation, and Sev= Severe)

Sample	Exteracellular matrix deposision	Vascularization (angiogenesis)	Foregin body gaint cell	Inflammation
PHB1	Mod	Mild	Mod	Mod
PHB2	Mod	Mod	Mild	Mod
PHB3	Sev	Mild	Mod	Mild
PHB4	Mod	Mild	Mod	Mod
PHB5	Mod	Mod	Sev	Mod
PHB/CNTs1	Mild	Mod	Mild	Mild
PHB/CNTs 2	Mild	Sev	Mild	Mild
PHB/CNTs 3	Mild	Sev	Mild	Mild
PHB/CNTs 4	Mild	Mod	Mod	Mild
PHB/CNTs 5	Mod	Mod	Mild	Mild

## Discussion

In the present study, we evaluated the in vitro and in vivo biocompatibility of pure PHB and PHB/1%CNTs composite scaffolds for PDL regeneration. First, we evaluated the structural and mechanical specifications in comparison with human PDL tissue. Presented SEM micrograph of electrospun fibers showed that the prepared fibers imitated the collagen fibrous structure in PDL, which consists of fibers with random orientation and connective pores that can provide a suitable space for the for the cells to proliferate and migrate. The mean fiber diameter in the composite scaffold has increased, which is about twice compared to pure PHB fibers. The definition of biomaterial is any material that can be used as a part of live system for a specific time that aims to treat, replace, or regenerate any organ or tissue. To produce a scaffold for tissue engineering applications, the biomaterial must have some requirements such as intrinsic biocompatibility, structural condition necessary for cell proliferation and migration, mechanical properties similar to the desired tissue [ 31 ]. However, most of the polymers are not eligible to provide the condition necessary to create the scaffold to be used in tissue regeneration [ 32 ]. This increment might be caused by viscosity effect. This phenomenon was common and it confirmed that viscosity is an effective parameter during electrospinning [ 33 ]. The increment of the fiber diameter by addition of the second material was reported in several studies [ 31 ]. The diameter of the scaffold pores is the only section of electrospinning method that is not easy to directly control. This can be indiscernibly controlled by obtaining smaller diameter fibers as smaller fibers result in smaller, more tightly packed pores [ 34 - 35 ]. However, it is not possible to alter the pore size without changing any of the other electrospinning parameters [ 33 ].

CNTs as the second material in the composite scaffold have led to improvement in the tensile strength. The mechanical properties of the scaffold must be adapted to the specific tissue to guarantee the required mechanical functions during the formation of the new tissue [ 31 ]. Given that the target tissue is PDL, the tensile strength of human PDL was more than that obtained for pure PHB. By addition of only 1% of CNTs, the tensile strength has been almost equal to PDL, which is suitable for PDL regeneration. The noticeable increment of the mechanical specifications by small amounts of CNTs (mainly less than 2%) has been reported in numerous studies [ 36 ].

The wettability of the composite scaffold was also increased in the presence of CNTs. According to Yuan, *et al*. [ 37 ] a contact angle less than 90° indicates that wetting of the surface is favorable, and the fluid will spread over a large area on the surface, while the contact angles greater than 90° generally means that wetting of the surface is unfavorable, so the fluid will minimize its contact with the surface and form a compact liquid droplet37. Functional groups of CNTs (COOH) increased the amount of oxygen on the surface, increasing the quantity of C-O [ 38 ]. This increment has caused a significant reduction of WCA value in the scaffolds. It can be concluded that this significant reduction of contact angle from 121◦ for pure scaffold to 86◦ for composite scaffold have been caused by the presence of CNTs in the scaffold.

The capability of a material to absorb sedimentary apatite on its surface from SBF solution is one of the ways that can be used to evaluate the bioactivity of a material. The essential requirement for a material to bond to the living bone is the formation of apatite on its surface in the living body and that this in vivo apatite formation can be reproduced in SBF [ 39 ]. Since the PDL tissue joins the cementum covering the root to the alveolar bone, the scaffolds for PDL regeneration should have the potential to be bonded with alveolar bone. There have been more sedimentary apatite crystals on the surface of composite scaffold compared to pure PHB scaffold. As a result, it could be concluded that CNTs within the scaffolds can increase the bioactivity and formation of HA crystals on the surface of the fibers (the ability of the material to bond with the living bone tissue). This might have been caused by hydrophobic nature of PHB (as obtained by contact angle study) because high hydrophobic surface prevents water absorption [ 40 ]. 

The primary biocompatibility tests can be done both in vitro and in vivo. In vitro tests evaluate the reaction of the cultured cells in the presence of the experimental material [ 41 ]. Because of the target tissue (PDL), human PDLSCs have chosen to evaluate in vitro biocompatibility of the scaffolds. The results showed that the presence of CNTs has caused more PDLSCs attachment on the composite scaffold surface. Less cell attachment on the surface of the pure PHB scaffold might be caused by the hydrophobic nature of PHB (Hydrophobic surfaces show poor cell adhesion) [ 42 ]. Many researchers have shown the improvement in the viability of different kinds of cells on the composite scaffolds in the presence of CNTs [ 43 - 45 ]. 

A primary and standard method to determine the in vivo biocompatibility of a material is subcutaneous implantation of the material using animal model and evaluation of the tissue response to the experimented material after a specific period [ 36 , 46 ]. In the present study, after 5 weeks of subcutaneous implantation, the tissue reaction was characterized at the area between the scaffold and surrounding connective tissue. Functionalized CNTs in the PHB scaffold have caused a reduction in the inflammatory cells, increased angiogenesis, and better tissue compatibility compared to pure PHB. These results were in the same line with those of other studies, which have shown lower inflammatory responses, achieved with CNTs in the composites. In terms of in vivo biocompatibility, many researchers have reported that CNTs in low amounts can improve the biocompatibility of the composite scaffolds [ 47 - 51 ]. Paiyz, *et al*. evaluated the effect of functionalized CNTs in the tissue compatibility of Poly (lactide-co-glycolide) (PLGA) scaffold and reported that PLGA/1%CNTs had a milder response than pure PLGA scaffold [ 47 ]. In another study, Eri *et al*. coated CNTs on the collagen scaffold for bone tissue engineering purpose and reported that CNT-coating of the collagen scaffold had favorable biocompatibility compared to uncoated scaffolds [ 48 ].

## Conclusion

PHB/1%CNTs scaffolds were successfully obtained by electrospinning that mimicked the fibrous connective tissue of PDL. The tensile strength of PHB/1%CNTs composite were near PDL and greatly improved compared to the pure PHB scaffold. Functionalized CNTs could improve the wettability, bioactivity, in vitro cell viability, and in vivo tissue compatibility of the scaffolds. According to the results, PHB/1%CNTs scaffold might have the potential to be used in periodontal tissue regeneration.
